# Effect of Organosilane Coupling Agents on Thermal, Rheological and Mechanical Properties of Silicate-Filled Epoxy Molding Compound

**DOI:** 10.3390/ma13010177

**Published:** 2020-01-01

**Authors:** Rok Šinkovec, Branka Mušič

**Affiliations:** Nanotesla Institut Logatec, Obrtna cona Logatec 4, 1370 Logatec, Slovenia; branka.music@kolektor.com

**Keywords:** epoxy molding compound, organosilane, silicate filler, tensile strength, thermal analysis, rheology

## Abstract

Global industries strive towards the production of materials with superior mechanical characteristics, and their development remains a big challenges. One of the more interesting materials that exhibit these properties are silicate-filled epoxy molding compounds (EMCs). A good interaction between silicate filler and epoxy matrix is generally needed to achieve advantageous mechanical properties, as well as the desirable rheological behavior of EMCs. Understanding the influence of different organosilane coupling agents on the rheological and mechanical properties of EMCs is essential in the development and optimization of the manufacturing process. For this matter, a mixture of calcium silicate and aluminosilicate was treated by using organosilane coupling agents with different chemical structures and thus treated silicates were applied as fillers in the EMCs. The thermal behavior of the organosilane-modified, silicate-filled EMCs was studied by using differential scanning calorimetry (DSC) and thermomechanical analysis (TMA). Flow-curing behavior (torque rheometer) and spiral flow length measurement (EMMI) were used to monitor the rheological properties and reactivity of the EMCs. The results showed that 3-glycidyloxypropyltrimethoxysilane- and 3-aminopropyltriethoxysilane-treated filler had a greater influence on the tensile strength of hot-pressed test samples, while 3-aminopropyltriethoxysilane and a blend of primary and secondary aminosilanes had a more significant impact on the rheological behavior of the material.

## 1. Introduction

Epoxy-based composites are known for their good thermal and dimensional stability, outstanding moisture and chemical resistance, and superior electrical and mechanical properties [1]. Therefore, they have found their use in many industrial fields, including electronic, automotive and construction. 

Different types of inorganic fillers, like kaolin [2], mica [3], silica [4], and calcium silicate [5,6], are mostly used to improve thermal stability and mechanical properties and to reduce the cost of final products in a wide variety of thermosetting composites. The common characteristic of these fillers is their hydrophilic surface, which makes it harder to achieve good wetting with the polymer. In order to overcome this problem, organofunctional silane coupling agents are used to modify the surface chemistry of the filler, thus improving filler–polymer interactions [7,8,9]. 

These compounds contain two types of functional groups: the silane reactive or hydrolysable groups, which exhibit affinity to the filler, and an organic chain, which terminates in a functional group with chemical affinity to the polymer [7,10]. One of the most common silane reactive groups is that of the alkoxy, which often must first undergo hydrolysis in order to form a stable covalent bond with the filler. They are most effective in combination with filler types with many reactive hydroxyl groups and an adequate amount of surface water, forming siloxane bonds with the substrate and the reactive organic chains to bond with the polymer [11]. This can provide a stable link between the polymer and the filler, with the possibility to enhance mechanical strength, thermal properties and chemical resistance.

In the past, various researchers have compared the effects of silane coupling agents on different properties of filled epoxy composites. Bajaj et al. studied the effect of two different organosilanes and an organo-zirconate on the thermal and electrical properties of mica/epoxy composites [12]. Chang et al. researched the influence of amine-modified silica nanoparticles on the thermal and mechanical properties of epoxy composites [13]. The research done by Yu et al. was focused on the microstructure, mechanical behavior, and heat resistant properties of organosilane-treated nanosilica/epoxy composites [14]. Ianchis et al. investigated the thermal and mechanical properties of epoxy nanocomposite with alkoxysilane-treated clay as a filler [15]. Piscitelli et al. examined the mechanical properties of montmorillonite-doped epoxy resins with different aminosilanes [16]. Jung-Hyun et al. studied the adhesion properties, flexural strength and thermal properties of epoxy molding compounds (EMCs) that were prepared from aminosilane-modified epoxy resin [17]. None of these studies included information about the influence of EMCs on rheology or flow-curing behavior, even though this influence is an important aspect for their industrial application. 

The aim of this work was to investigate the advantages of different organosilane coupling agents on the end characteristics of EMCs that are meant for industrial utilization, specifically for the encapsulation and overmolding of electronic components. The focus was set on the thermal (glass transition temperature—T_g_) and mechanical properties (tensile strength) of calcium silicate/aluminosilicate-filled EMCs, as well as their rheological properties (flow-curing behavior and spiral flow length).

## 2. Materials and Methods 

### 2.1. Materials

**Figure 1:**
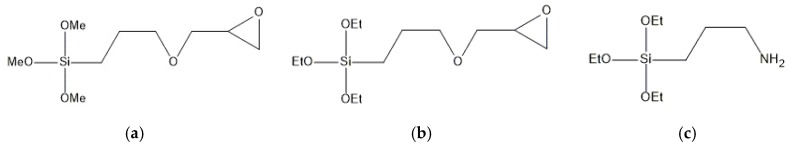
Skeletal structural formulas of the organosilane coupling agents tested in this study: (**a**) 3-glycidyloxypropyltrimethoxysilane; (**b**) 3-glycidyloxypropyltriethoxysilane; and (**c**) 3-aminopropyltriethoxysilane.

All the materials used in this study are commercially available and were used as such. The resin used was an epoxidized *o*-cresol novolac resin with average epoxy equivalent weight (EEW) of 210 g/eq. A phenolic novolac resin with a hydroxyl equivalent weight (OHeq) of 105 g/eq and a combination of 2-methylimidazole/dicyandiamide (Sigma-Aldrich, St. Louis, MO, USA) were used as a hardener and a curing accelerator, respectively. The amount of the binding epoxy-novolac system in the composite was 25 wt.%. Combined aluminosilicate and calcium silicate fillers in a weight ratio 1:0.7 (up to 25 wt.%) were treated with the same amount (3.7 wt.%) of different organosilane coupling agents (Evonik Industries AG, Essen, Germany; Figure 1). Where commercially available 3-aminopropyltriethoxysilane-treated calcium silicate was used, no additional organosilane coupling agent was added. Other components used were glass beads and glass fibers, pigment, and a mold release agent.

### 2.2. Properties of Organosilane Coupling Agents

Table 1 summarizes the chemical names, viscosities and pH values of all four organosilanes that were investigated in this study. The properties of said silanes are as follows:

(1) 3-glycidyloxypropyltrimethoxysilane: This type of silane has a reactive organic epoxy group and hydrolysable inorganic methoxysilyl groups, thus allowing it to function as an adhesion promoter or surface modifier. The nature of 3-glycidyloxypropyltrimethoxysilane allows it to chemically bind to both an inorganic filler and a resin matrix.
(2) 3-glycidyloxypropyltriethoxysilane: In addition to the 3-glycidyloxypropyltrimethoxysilane, this organosilane possesses an organic epoxy group on one end and inorganic ethoxysilyl groups on the other. The main difference is in the rate of hydrolysis of the alkoxysilyl groups, as the ethoxy groups hydrolyze at a slower rate than methoxy groups.
(3) 3-aminopropyltriethoxysilane: Based on the primary 3-aminopropyl group, this compound is widely used for the surface treatment of fillers, such as wollastonite and mica. It is a versatile organosilane that is used in epoxy, phenolic and thermoplastic polymeric composites. The amine group is reactive towards the epoxy groups of the resin and can have an influence on the curing reaction.
(4) Proprietary aminosilane composition: This commercially available aminosilane composition consists of primary and secondary aminosilanes, of which 90% is 3-aminopropyltriethoxysilane.

### 2.3. Preparation of the EMCs

To aluminosilicate and calcium silicate fillers, different organosilane coupling agents (Table 2), were added and thoroughly mixed for 5 min in a high-speed container mixer (Mixaco Lab CM 10-SM, Mixaco Maschinenbau Dr. Herfeld GmbH & Co. KG, Neuenrade, Germany). Epoxy and phenol novolac resins were weighed out according to a correct equivalent ratio of EEW:OHeq = 1:1. Glass beads, the curing accelerator, the mold release agent, and pigment were added, and the mixture was homogenized by using a container mixer (Mixaco CM 50 MT, Mixaco Maschinenbau Dr. Herfeld GmbH & Co. KG, Neuenrade, Germany). The mixture was fed to an axial oscillating, continuous single screw kneader (Buss PLK 46-11R, Buss AG, Pratteln, Switzerland) and kneaded at temperatures from 110 to 120 °C. During the kneading process, glass fibers were added in a continuous fashion. The extruded material was passed through a cooled two-roll calender (Kolektor iDrium, Ljubljana, Slovenia), air-cooled, and crushed on a power cutting mill (Pulverisette 25, Fritsch, Idar-Oberstein, Germany). The obtained granulate samples, with particle size distribution from 3 to 5 mm, were marked as S1–S5.

**Figure 2:**
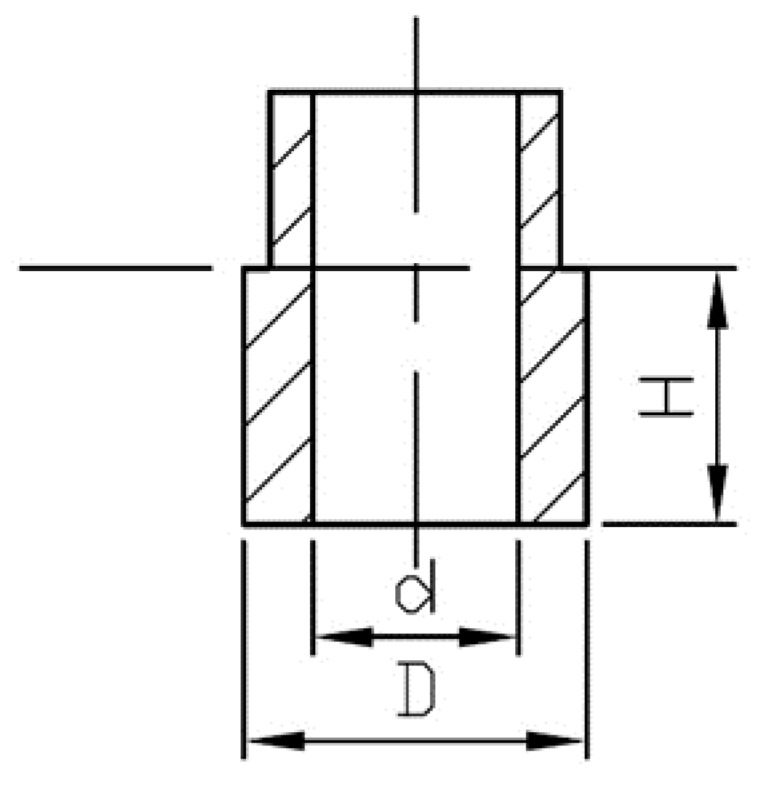
Technical drawing of molded test samples for tensile strength measurements.

EMC granulate samples were hot pressed into hollow cylinder-shaped test samples, marked AM1–AM5, by using a hydraulic hot press at temperature of molding tool 175 °C ± 3 °C. The molding cycle was prearranged to 195 seconds, after which the test samples were left to cool down to ambient temperature. The bottom part (H × D × d = 15 × 20 × 12 mm^3^, Figure 2) was cut off with an angle grinder, and the cylinders were subjected to post-curing treatment in a laboratory fan oven for 5 h at 210 °C and marked PC1–PC5.

### 2.4. Testing Methods

Five different EMCs were prepared and tested, each with a different organosilane coupling agent and marked as presented in Table 2. The amount of applied organosilane was equal in all samples (3.7 wt.%), with the exception of S3, where calcium silicate was pre-treated with 3-aminopropyltriethoxysilane by the manufacturer. The weight fraction of the other components remained consistent. The methods for analyzing the EMCs properties are as follows.

- Differential scanning calorimetry (DSC): The thermal behavior of the EMCs was analyzed with a thermal analyzer (DSC823^e^ Module STAR^e^ System, Mettler Toledo, Greifensee, Switzerland). The measurements of samples (10–15 mg) were performed in a 40 µL aluminum pan with pierced lid from 25 to 300 °C at a heating rate 10 K min^−1^ in an air atmosphere. These conditions were used to investigate the heat flow profile of the granulate after the molding and post-curing of the samples.
- Thermomechanical analysis (TMA): The glass transition temperature (T_g_) was determined with thermomechanical analysis (Mettler Toledo TMA/SDTA840, Mettler Toledo, Greifensee, Switzerland). The test samples were cut to dimensions of 10 mm × (3.5 ± 0.5 mm), and the measurement was performed at a heating rate of 10 K min^−1^ from 30 to 300 °C with the use of a 3 mm probe with flat end (0.05 N force) under air atmosphere.
- Rheological properties: The flow-curing behavior of the EMCs was determined with a torque rheometer with a pneumatic loading system (Brabender Plastograph EC, Mixer MB30, Brabender, Duisburg, Germany). The sample mass was 30 g, the blade speed 45 rpm, and the temperature of the mixing bowl was 150 °C. Measurements were evaluated with Brabender Data Correlation software, according to standard DIN 53 764 [18].
- Spiral flow length was measured on a transfer molding press (Lauffer UVKO 25, Horb am Neckar, Germany) at a tool temperature of 150 ± 3 °C and a transfer pressure of 69 bar, according to the standard ASTM D3123–09(2017) [19]. The molding cycle was set to 285 s, after which the spiral was removed from the mold and measured. The method is used to study the behavior of molding compounds under specific conditions and is widely used in the flow supervision of EMCs in industrial environments.
- Mechanical test: Tensile strength characterization was carried out after the post-curing treatment of the cylindrical test samples. The sample was mounted onto the working platform, and the measurement was carried out at a speed of 86 mm min^−1^ by using a static tensile machine (Z100, Zwick/Roell, Ulm, Germany).

## 3. Results and Discussion 

### 3.1. Thermal Analysis

**Figure 3:**
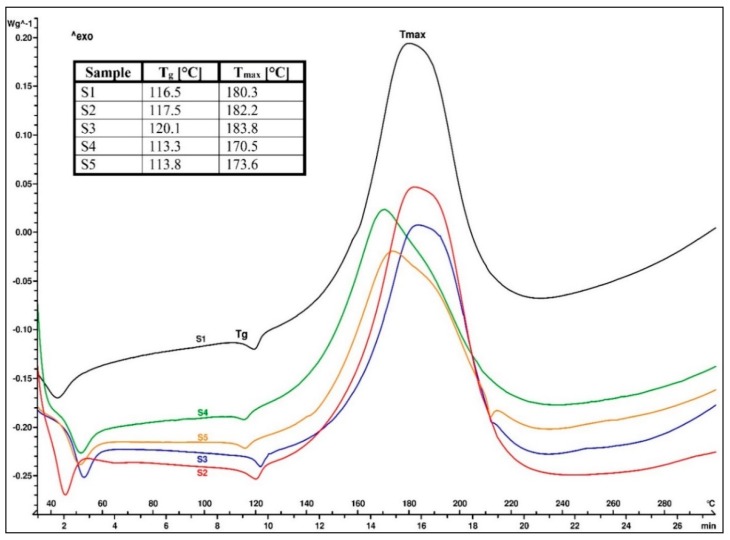
Differential scanning calorimetry (DSC) thermogram of epoxy molding compound (EMC) granulates.

The DSC analysis was performed in order to evaluate the thermal behavior of each EMC sample that was prepared with a different organosilane coupling agent. The thermograms of the EMC granulate samples, marked S1–S5, are presented in Figure 3. The first endothermic peak in the range of 45 to 55 °C represented the softening point of the epoxy resin, while the second, smaller endothermic peak spanning from 115 to 125 °C was the glass transition temperature (T_g_) of the EMC granulate samples. An exothermic cross-linking reaction between the epoxy and phenolic novolac resins started immediately after T_g_ and was visible between 140 and 220 °C. Above 220 °C, the thermal decomposition of all five EMC samples could be detected. In our case, different organosilane coupling agents affected T_g_ and the peak temperature of the cross-linking reaction (T_max_). The endothermic peak that was present on the heat flow curve at 210 °C in the S3 and S5 samples represented the melting of the unreacted curing accelerator [20].

**Figure 4:**
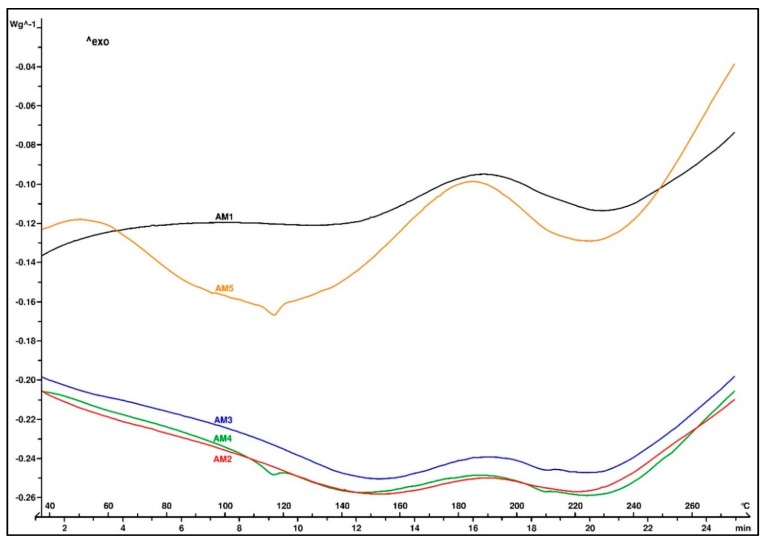
DSC thermogram of the molded EMC test samples.

Figure 4 illustrates the thermal profile of the molded EMC samples. All samples showed similar behavior in the DSC analysis, with a heat flow increase in the range from 160 to 220 °C, suggesting that the material was not completely cured. In case of the added 3-aminopropyltriethoxysilane in the AM4 sample, the T_g_ of the molded composite was still visible on the thermogram. This was also true in the case of AM5, where the coupling agent was a blend of primary and secondary aminosilanes, indicating that the molding temperature was too low for that type of EMC.

**Figure 5:**
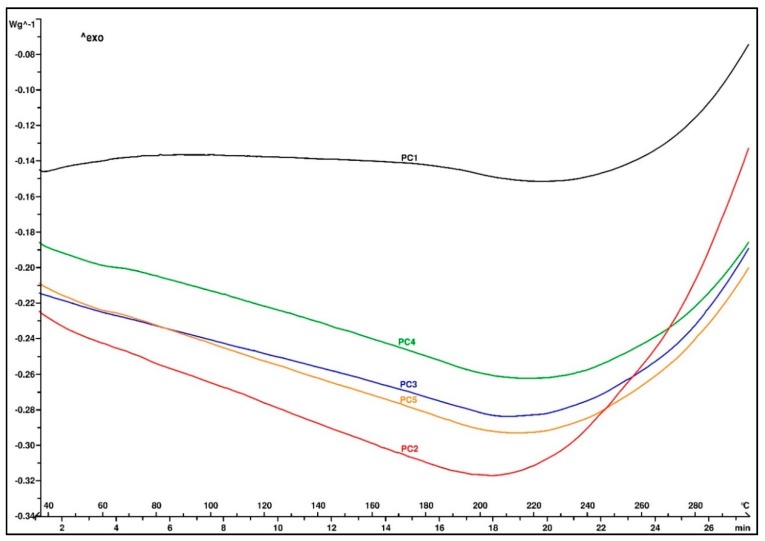
DSC determination of decomposition temperature.

A thermogram of the samples after the post-curing treatment is shown in Figure 5. No residual exothermic peak of the cross-linking reaction was observed, indicating that the material was completely cured. The thermal decomposition started above 200 °C and showed the good thermal stability of all five EMCs.

**Figure 6:**
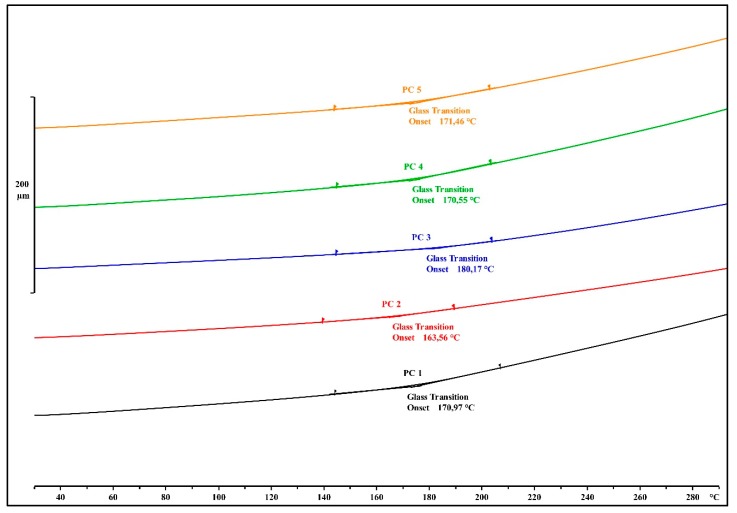
Glass transition temperature (T_g_) determination of the EMC samples.

The impact of the different chosen organosilane coupling agents on the T_g_ of the EMCs was analyzed with TMA after the post-curing treatment (Figure 6), and the measured values are presented in Table 3.

The commercially available 3-aminopropyltriethoxysilane-treated calcium silicate displayed the highest T_g_ of 180 °C in the post cured PC3 sample. In comparison to the EMCs with 3-glycidyloxypropyltrimethoxysilane and 3-glycidyloxypropyltriethoxysilane, the T_g_ was greater by 10 and 17 °C, respectively. This can be attributed to the fact that the bonds between the 3-aminopropyltriethoxysilane and the filler were stronger due to different organosilane application procedures. EMCs that were prepared with 3-aminopropyltriethoxysilane and with a blend of primary and secondary aminosilanes displayed a T_g_ of 170.5 and 171.5 °C, respectively (Table 3). 

With our selection of organosilane coupling agents, we were able to manipulate the Tg of the EMCs, which is one of the more important parameters in EMC production.

### 3.2. Rheological Behavior of EMCs

**Figure 7:**
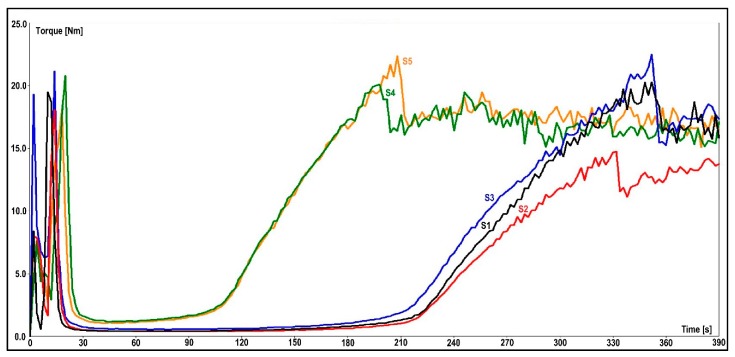
Flow-curing behavior of the EMCs.

(1) The flow-curing behavior of the EMCs could be determined from measurements with the torque rheometer. The plastogram exhibits torque measurement over a time period and shows structural changes in the material. The presented values of this type of measurement are residence time (tV), minimum torque (B), and reaction time (tR). The residence time determines the duration of the material in its molten state before the curing reaction starts. The torque minimum defines the melt viscosity of a test sample before curing. A lower torque value means a lower viscosity of the molten test sample and vice versa. After reaching the maximum peak, torque stabilizes, thus showing the end of the curing reaction. The time it takes for a material to reach that value is determined by the curing time. The combined plastograms are depicted in Figure 7, and all the significant values that were gathered with this measurement are presented in Table 4.

As seen from the results in Table 4, the 3-aminopropyltriethoxysilane in S4 shortened the cross-linking reaction time in comparison to the S1–S3 samples by almost 50%. A similar behavior was observed when a blend of primary and secondary aminosilanes was used in the S5 granulate, thus indicating that the presence of an amino group in the two organosilanes contributed to the curing reaction of the epoxy resin. Despite the fact that the S3 granulate was prepared with commercially available 3-aminopropyltriethoxysilane-treated calcium silicate, it showed different flow-curing behavior than the S4 and S5 samples. The reason for this lies in the different procedures for the application of 3-aminopropyltriethoxysilane to the filler that were used by the manufacturer. 

(2) Spiral flow length (EMMI-Epoxy Molding Materials Institute) depends on a multitude of factors, including the tool temperature, injection pressure, melt viscosity, and composition of the material. In general, if we keep the temperature and injection pressure constant, the material with the lower melt viscosity will travel a greater length in the mold and will consequently have a longer spiral flow length. EMCs with either 3-glycidyloxypropyltrimethoxysilane or 3-glycidyloxypropyltriethoxysilane gave higher spiral flow length values than the EMCs prepared with 3-aminopropyltriethoxysilane. The spiral flow length was reduced by almost 50% when 3-aminopropyltriethoxysilane was added as a coupling agent (Table 5). This observation was expected from the flow-curing behavior measurements and could be attributed to the fact that epoxysilanes did not contribute to the curing reaction of the epoxy-novolac binding system. Furthermore, they also lowered the melt viscosity of the material, thus reducing its flow resistance and allowing it to flow more easily. 

### 3.3. Tensile Strength

Table 6 summarizes the tensile strength determination of the molded test samples that were prepared from the EMCs. From the presented data, we noticed a difference in the force measurement values in regard to the different organosilane coupling agents applied.

The biggest effect on tensile strength came from the addition of 3-glycidyloxypropyltrimethoxysilane to the inorganic filler, as seen in the PC1 sample. This could be associated with the fact that during the reaction, the epoxy ring opened and sped up the hydrolysis of the methoxy substituents, therefore improving the bonding with the filler [21]. The PC2 sample from the EMC that was prepared with 3-glycidyloxypropyltriethoxysilane showed a lower tensile strength, possibly due to the slower hydrolysis of the ethoxy group. This lower hydrolysis rate affected the ability of organosilane to form strong bonds with the filler, resulting in a decrease of tensile strength. Adding 3-aminopropyltriethoxysilane or a commercially available blend of primary and secondary aminosilanes resulted in a slight drop of the tensile strength values compared to the sample with 3-glycidyloxypropyltrimethoxysilane. The decrease was from 5% to 10%, except in the case of commercially 3-aminopropyltriethoxysilane-treated calcium silicate and the 3-glycidyloxypropyltriethoxysilane-treated filler. In the PC2 and PC3 samples that were prepared with these two treated fillers, the tensile strength was decreased by 18% and 23%, respectively, in comparison to PC1. 

## 4. Conclusions

The results presented in this study show that the choice of organosilane coupling agent has a significant influence on the melt viscosity and reactivity of EMCs, as well as the mechanical properties of the molded test samples. The findings are as follows:

A slight difference in the T_g_ of the fully cured material was noticed (164–171 °C), with commercially available 3-aminopropyltriethoxysilane-treated calcium silicate giving the highest value (180 °C). 

The flow-curing behavior of the composite samples indicated a faster curing reaction with the addition of an aminosilane to the silicate filler in regard to epoxysilane. The melt viscosity value of the EMC with the added aminosilane was two to three times greater as for the EMC with the added epoxysilane. 

The highest spiral flow length of EMCs was observed when 3-glycidyloxypropyltrimethoxysilane was added. The spiral flow length of the EMCs decreased as follows: epoxysilane-treated calcium silicate > commercially available aminosilane-treated calcium silicate > aminosilane-treated calcium silicate.

The best tensile strength was achieved when 3-glycidyloxypropyltrimethoxysilane was added to the inorganic filler. The decrease of the tensile strength values of EMCs was from 5% to 10% when an aminosilane was used instead, while the commercially available aminosilane-treated calcium silicate gave the worst results. 

An overall comparison demonstrated that with different chemical structures of organosilanes, we can manipulate the thermal, rheological and mechanical properties of EMCs. One of the industry’s requirements is the right rheological behavior to achieve the optimal number of products that are formed in a time unit. An EMC prepared with the addition of 3-aminopropyltriethoxysilane to the filler maintains a quite high tensile strength, while its cross-linking reaction time is reduced by half. 3-aminopropyltriethoxysilane thus leads to a reduction of production costs. For this purpose, the outcome of these findings should be tested in an actual injection or transfer molding process for industrial applications and should be further adjusted to the industry’s specific needs.

## Figures and Tables

**Table 1:** Organosilane-type coupling agents with their corresponding properties.

Organosilane	Chemical Name	Viscosity at 25 °C, ^1^ mPa·s	pH ^1^
1a	3-glycidyloxypropyltrimethoxysilane	3.65	7.5
1b	3-glycidyloxypropyltriethoxysilane	3.35	4.0
1c	3-aminopropyltriethoxysilane	1.85	11.3
1d	Blend of primary and secondary aminosilanes	2.10	11.3

^1^ Data provided by the supplier.

**Table 2:** Sample markings in regard to applied organosilane coupling agents.

Organosilane Mark	Sample Marking		
	Granulate	After Molding	Post Curing
1a	S1	AM1	PC1
1b	S2	AM2	PC2
1c ^2^	S3	AM3	PC3
1c	S4	AM4	PC4
1d	S5	AM5	PC5

^2^ Commercially available 3-aminopropyltriethoxysilane-treated calcium silicate.

**Table 3:** Glass transition temperatures, depending on applied organosilane.

Organosilane Mark	Sample Mark	Tg, °C
1a	PC1	170.97
1b	PC2	163.56
1c	PC3	180.17
1c	PC4	170.55
1d	PC5	171.46

**Table 4:** Flow-curing behavior properties in regard to different organosilanes.

Organosilane Mark	Sample Mark	Torque Minimum (B), Nm	Residence Time (tV), s	Reaction Time (tR), s
1a	S1	0.4	209	305
1b	S2	0.4	203	288
1c	S3	0.6	200	307
1c	S4	1.2	87	154
1d	S5	1.1	91	168

**Table 5:** EMMI-Epoxy Molding Materials Institute spiral flow length measurements.

Organosilane Mark	Sample Mark	Spiral Flow Length, Inch
1a	S1	36.5 ± 1.3
1b	S2	38.5 ± 0.2
1c	S3	28.4 ± 2.0
1c	S4	19.6 ± 1.0
1d	S5	22.0 ± 1.1

**Table 6:** Tensile strength measurement results.

Organosilane Mark	Sample Mark	Tensile Strength, N
1a	PC1	2110 ± 30
1b	PC2	1740 ± 40
1c	PC3	1640 ± 10
1c	PC4	2000 ± 40
1d	PC5	1900 ± 40
